# Effect of Prior Influenza A(H1N1)pdm09 Virus Infection on Pathogenesis and Transmission of Human Influenza A(H5N1) Clade 2.3.4.4b Virus in Ferret Model

**DOI:** 10.3201/eid3103.241489

**Published:** 2025-03

**Authors:** Xiangjie Sun, Jessica A. Belser, Zhu-Nan Li, Nicole Brock, Joanna A. Pulit-Penaloza, Troy J. Kieran, Claudia Pappas, Hui Zeng, Jessie C. Chang, Paul J. Carney, Brandon L. Bradley-Ferrell, James Stevens, Terrence M. Tumpey, Min Z. Levine, Taronna R. Maines

**Affiliations:** Author affiliation: Centers for Disease Control and Prevention, Atlanta, Georgia, USA

**Keywords:** Influenza A virus, A(H5N1), influenza, respiratory infections, zoonoses, viruses, clade 2.3.4.4b, prior infection, pathogenesis, transmission, ferrets

## Abstract

Reports of human infections with influenza A(H5N1) clade 2.3.4.4b viruses associated with outbreaks in dairy cows in the United States underscore the need to assess the potential cross-protection conferred by existing influenza immunity. We serologically evaluated ferrets previously infected with an influenza A(H1N1)pdm09 virus for cross-reactive antibodies and then challenged 3 months later with either highly pathogenic H5N1 clade 2.3.4.4b or low pathogenicity H7N9 virus. Our results showed that prior influenza A(H1N1)pdm09 virus infection more effectively reduced the replication and transmission of the H5N1 virus than did the H7N9 virus, a finding supported by the presence of group 1 hemagglutinin stalk and N1 neuraminidase antibodies in preimmune ferrets. Our findings suggest that prior influenza A(H1N1)pdm09 virus infection may confer some level of protection against influenza A(H5N1) clade 2.3.4.4.b virus.

Highly pathogenic avian influenza A(H5N1) clade 2.3.4.4b viruses have exhibited rapid global spread in wild birds since 2021 (*1*,*2*). That spread has resulted in numerous outbreaks in domestic poultry and sporadic infections in nonavian hosts (*1*,*2*), including >20 mammal species in the United States alone since January 2022 (*3*). On March 25, 2024, H5N1 clade 2.3.4.4b virus was reported in dairy cows in Texas and Kansas (*4*), and the virus was subsequently detected in dairy herds from over a dozen states within 5 months (*5*). Those outbreaks resulted in several confirmed human infections, primarily after exposure to infected dairy cows or poultry (*6*). Although human cases from 2024 generally exhibited mild symptoms (*7*,*8*), clade 2.3.4.4b viruses can cause severe infection in humans (*9*). Viruses isolated before and concurrent with ongoing dairy farm outbreaks possess an efficient capacity for replicating in mammalian hosts and are capable of systemic spread and lethal infection in both mouse and ferret models (*10*–*13*). Furthermore, some clade 2.3.4.4b viruses, including viruses isolated from dairy farm workers in 2024, exhibit a limited capacity for airborne transmission between ferrets, suggesting a heightened risk to public health (*10*–*12*,*14*). Those incidents highlight the ongoing threat posed by H5N1 viruses and underscore the need for comprehensive risk assessments to evaluate the capacity of clade 2.3.4.4b viruses to cause infection and disease in diverse human populations.

Although almost all influenza A(H5N1) human cases in the United States have been associated with mild disease, and no human-to-human transmission has been reported, assessing whether the human population has cross-reactive antibodies that can provide protection against the disease caused by H5N1 virus infection is critical. Despite previous serologic surveys indicating very low (0%–1.9%) seropositivity against H5N1 virus in the general population (*15*), recent findings have shown relatively high levels of cross-reactive neuraminidase inhibition antibodies to H5N1 clade 2.3.4.4b viruses in healthy adults, likely because of prior exposure to influenza A(H1N1)pdm09 (pH1N1) virus (*16*). In addition, broadly reactive hemagglutinin (HA) stalk antibodies induced by seasonal influenza A(H1N1) and A(H3N2) virus infections in humans have been suggested to provide some level of protection against zoonotic H5N1 and H7N9 virus infections (*17*,*18*).

Well-controlled studies in mammalian models can provide crucial insight into the ability of prior influenza A virus (IAV) exposure to modulate subsequent disease after homologous or heterologous viral challenge (*19*). Cross-protection against H5N1 clade 1 viruses induced by prior human seasonal IAV infection has been previously shown in the ferret model (*20*,*21*). However, those studies have not assessed the role of preexisting immunity in modulating transmission outcomes and have not explored the extent of cross-protection or the specific types of cross-reactive antibodies in preimmune animals. We used a ferret model to evaluate viral replication and transmission of the H5N1 clade 2.3.4.4b virus in ferrets with or without prior immunity against pH1N1 virus. 

## Materials and Methods

### Viruses

We propagated stocks of 3 virus strains: pH1N1 A/Nebraska/14/2019 (Neb/14); highly pathogenic H5N1 clade 2.3.4.4b A/Texas/37/2024 (Texas/37); and low pathogenicity H7N9 A/Anhui/1/2013 (Anhui/1), which was isolated from the first wave of human infections in China in 2013, as described previously (*11*,*22*,*23*). All virus preparation and animal infection experiments were conducted in Biosecurity Level 3 containment laboratories including enhancements required by the US Department of Agriculture and the Federal Select Agent Program. All animal studies were approved by the Institutional Animal Care and Use Committee of the Centers for Disease Control and Prevention (CDC) and were conducted under the guidance of the CDC’s Institutional Animal Care and Use Committee in an AAALAC-accredited animal facility.

### Primary Intranasal Infection with pH1N1 Virus

We serologically tested twelve 8-month-old ferrets by using the 2023–2024 World Health Organization influenza reagent kit; all tested negative for circulating influenza viruses, including A/Victoria/2570/2019 (H1N1)pdm09, A/Delaware/01/2021 (H3N2), B/Phuket/3073/2013 (Yamagata lineage), and B/Michigan/01/2021 (Victoria lineage). We then intranasally inoculated the ferrets with >10^2^ PFU Neb/14 virus per ferret in 1 mL phosphate-buffered saline under ketamine/xylazine anesthesia. All 12 ferrets developed productive infection and exhibited hemagglutination inhibition (HI) titers of 80–320 against homologous virus by day 93 postinoculation. We termed that group of ferrets as preimmune.

### Ferret Inoculation and Evaluation of Viral Replication and Transmission

We then inoculated groups of the preimmune ferrets and naive ferrets by respiratory inhalation of aerosolized virus for 15 minutes, as previously described (*24*), and used an elevated relative humidity (≈65%) to better emulate environmental conditions on dairy farms. We administered doses varying from 10^0.5^ to 10^2.8^ PFU (Table). At 24 hours postinoculation, we pair-housed groups of 3 naive or preimmune ferrets with inoculated ferrets at a 1:1 donor:contact ratio to assess virus transmission in a direct contact setting. We observed all ferrets daily for clinical signs of infection, including temperature rise, weight loss, and lethargy, and collected nasal wash daily until day 3 postinoculation for Texas/37 or day 4 postinoculation for Anhui/1 virus, as previously described (*11*,*22*), at which time we humanely euthanized inoculated animals to evaluate virus replication and systemic spread. We monitored contact ferrets for virus shedding and seroconversion at day 21 postinoculation as determined by HI titers using 0.5% turkey red blood cells. We determined productive transmission by detection of infectious virus in nasal wash specimens and seroconversion to homologous challenge virus.

**Table:** Pathogenicity and transmissibility of influenza viruses in naive and preimmune ferrets in study of the effect of prior influenza A(H1N1)pdm09 virus infection on pathogenesis of human influenza A(H5N1) clade 2.3.4.4b virus in ferret model*

Challenge virus				Inoculated animals‡						Contact animals	
	Status†			Dose, PFU§	Lethargy¶	Temp. rise, °C#	% Weight loss**	Peak titer (SD)††		Peak titer (SD)††	Transmission/ inoculation‡‡
	Donor	Contact									
Texas/37	Naive	Naive		10^0.5^	1.5	2.4 (2/2)	7.3 (2/2)	5.5 (0.7)		2.5 (0.6)	2/2
Texas/37	Naive	Preimmune		10^2.4^	1.9	1.7 (3/3)	7.9 (3/3)	4.2 (0.4)		NA	0/3
Texas/37	Preimmune	Naive		10^2.4–2.5^	1.7	1.5 (2/3)	5.5 (2/3)	2.5 (1.8)		NA	0/3
Anhui/1	Naive	Naive		10^2.7^	1.2	1.9 (2/3)	4.7 (3/3)	5.7 (0.8)		6.1 (1.5)	3/3
Anhui/1	Naive	Preimmune		10^2.8^	1.1	1.5 (2/3)	5.4 (1/3)	6.5 (0.4)		5.4 (0.8)	3/3
Anhui/1	Preimmune	Naive		10^2.6^	1.1	1.6 (2/3)	5.4 (2/3)	6.3 (0.2)		3.3 (1.5)	2/3

*Preimmune ferrets were those inoculated with influenza A(H1N1)pdm09 (A/Nebraska/14/2019). Naive and preimmune ferrets were challenged with highly pathogenic influenza A(H5N1) clade 2.3.4.4b A/Texas/37/2024 (Texas/37) and low pathogenicity influenza A(H7N9) A/Anhui/1/2013 (Anhui/1). NA, not applicable because titers were below the limit of detection; temp., temperature. †Ferrets were serologically naive to contemporary influenza A viruses (naive) or inoculated with A/Nebraska/14/2019 influenza A(H1N1)pdm09 virus 3 months before use in this study (preimmune). ‡Inoculated ferrets were monitored for clinical signs of disease for 3 days for the Texas/37 virus and 4 days for the Anhui/1 virus. All animals that were productively infected after aerosol exposure are shown (n = 3 for all groups except Texas/37 naive donor/naive contact, which is n = 2). §Presented dose of virus to ferrets following 15-min inhalation exposure to aerosolized virus. ¶Relative inactivity index of ferrets inoculated with the challenge virus specified. #Values in parentheses are no. affected/no. inoculated. Mean maximum temperature rise >1°C among all inoculated animals in degrees centigrade; number of ferrets with temperature rise >1°C in all productively infected ferrets was shown in parentheses. **Values in parentheses are no. affected/no. inoculated. Mean maximum weight loss >1% among all inoculated animals expressed as a percentage; number of ferrets with weight loss >1% in all productively infected ferrets was shown in parentheses. ††Mean maximum nasal wash titer +SD reported as log_10_ PFU/mL among ferrets with positive virus shedding; for NA, titers for all ferrets were below limit of detection of 10 PFU. ‡‡Values are no. affected/no. inoculated. Transmission was defined as both the detection of productive virus shedding and seroconversion of surviving animals to homologous challenge virus or confirmed infection requiring euthanasia. Two contact ferrets, each co-housed with a ferret productively infected with the Texas/37 virus, were humanely euthanized on days 3 and 4 postcontact; all contact ferrets co-housed with ferrets inoculated with Anhui/1 virus survived through day 21 postcontact.

All samples collected for infectious virus determination were frozen at −80°C until titration by standard plaque assay in Madin-Darby canine kidney cells (limit of detection 10 PFU/mL or g of tissue). We used a 2-way analysis of variance test in Prism 7.05 (GraphPad Software Inc., https://www.graphpad.com) to assess viral titer differences between naive and preimmune groups. We considered p<0.05 statistically significant.

### Serologic Assessment of Cross-Reactive Antibodies after Primary pH1N1 Virus Challenge

We developed and performed a high-throughput multiplex influenza antibody detection assay, as described previously (*25*,*26*). In brief, we included HA globular head, HA stalk, neuraminidase (NA), and nucleoprotein (NP) antigens from IAVs and HA globular head from influenza B viruses in this study (Appendix 1 Table). We obtained antigens from Sino Biological U.S. Inc. (https://www.sinobiological.com), the International Reagent Resource (https://www.internationalreagentresource.org), or by using an in-house baculovirus expression system to express and purify our own reagents (*27*,*28*). 

To each well of a black-wall 96-well plate, we added 50 µL of microsphere suspension containing 2,000 microspheres for each of the 32 bead regions in an assay buffer comprising 1× PBS with 0.05% Tween 20, 1% bovine serum albumin (BSA), and 0.5 mol sodium chloride (total 64,000 microspheres/well). We then added 1:200 diluted naive or preimmune ferret serum in assay buffer to appropriate wells in duplicates, including 2 serum pools on each plate as intraassay and interassay controls. We incubated plates in the dark at room temperature for 60 minutes on an orbital shaker, then washed plates 3 times with 100 μL of assay buffer by using Bio-Plex Handheld Magnetic Washer (Bio-Rad Laboratories, https://www.bio-rad.com). We then added 100 µL of protein A–phycoerythrin conjugate and incubated plates in the dark at room temperature for 60 minutes on an orbital shaker. Then, we washed plates 3 times with 100 μL of reading buffer comprising 1× PBS with 0.05% Tween 20, 1% BSA and read the plates by using a Bio-Plex MAGPIX Multiplex Reader (Bio-Rad Laboratories). We calculated median fluorescence intensities by using GraphPad 7.05 software (GraphPad Software Inc.).

## Results

### Cross-Reactive Antibodies Induced by pH1N1 Virus Infection

**Figure 1:**
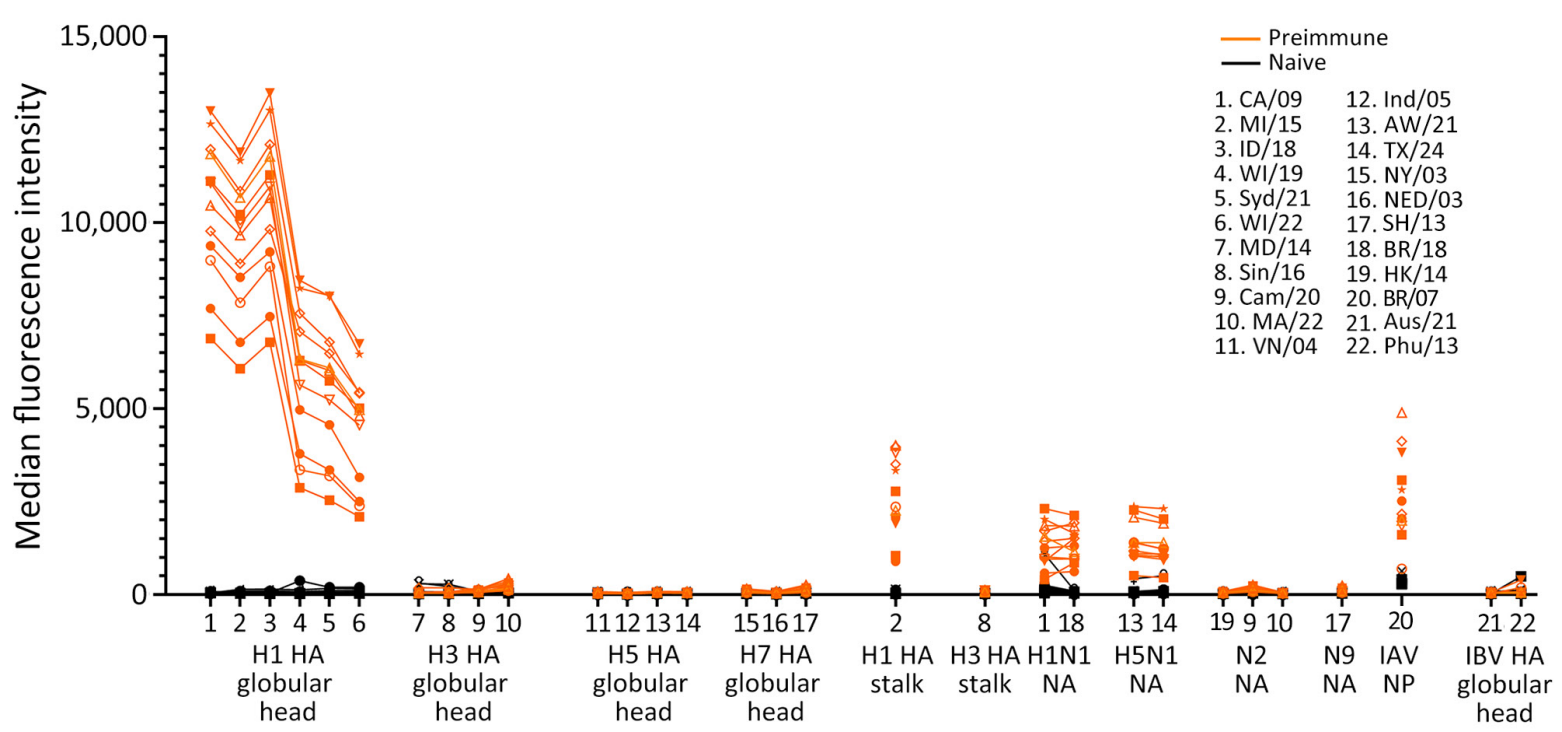
Detection of cross-reactive antibodies in study of the effect of prior influenza A(H1N1)pdm09 virus infection on pathogenesis and transmission of human influenza A(H5N1) clade 2.3.4.4b virus in ferret model. After primary pH1N1 infection, we detected cross-reactive antibodies by using a high-throughput multiplex influenza antibody detection assay. Serum samples from naive and preimmune ferrets were prediluted 200-fold and added to plates containing antigen-coated microspheres, then plates were incubated with protein A–phycoerythrin conjugate. Reported values represent the mean of duplicate assays. Antibody titers were expressed as median fluorescence intensity. Full virus strain names and sources of all antigens are provided (Appendix 1 Table). HA, hemagglutinin; IAV, influenza A virus; IBV, influenza B virus; NA, neuraminidase; NP, nucleoprotein.

We tested serum samples collected 93 days after primary pH1N1 virus infection for reactivity against various HA, NA, and NP antigens (Figure 1). We found the highest serum antibody levels detected were against the HA globular head from pH1N1 viruses predating the challenge strain, but we consistently detected antibodies against all 6 H1 HA targets assessed. We also detected antibodies reactive to the N1 NA, which were derived from either H1N1 or H5N1 virus isolates, including Texas/37, in all preimmune animals. Moreover, we detected antibodies against NP protein of pH1N1 virus A/Brisbane/10/2007 in preimmune ferrets. The NP proteins between Texas/37 and Neb/14 viruses share 94% similarity. However, we detected no antibodies against the HA globular head of H3, H5, or H7 viruses, or the NA from H3N2 or H7N9 viruses. In addition, Neb/14 virus infection induced antibodies reactive to group 1, but not group 2, HA stalks (Figure 1). Furthermore, alignments of surface exposed residues in the HA, including HA1 head and HA2 stalk, and NA proteins of Neb/14 and Texas/37 virus revealed higher similarities in NA surface residues (83.4%, 186/223) and in the HA2 stalk (80.4%, 111/138) compared with HA1 surface exposed residues (45%, 107/238) (Appendix 2 Figures 1). That finding supports the observed cross-reactivity to the HA2 stalk and Texas/37 NA but not to the Texas/37 HA head in preimmune ferrets. Our serologic findings demonstrated that pH1N1 infection can induce low levels of cross-reactive antibodies to H5N1 NA and the HA stalk from the group 1 H5N1 but not to group 2 H7N9, virus where cross-reactive antibodies remained at undetectable levels.

### Attenuation of Texas/37 Virulence and Transmission in pH1N1 Preimmune Ferrets

To assess if cross-reactive antibodies elicited in ferrets after pH1N1 infection could reduce H5N1 clade 2.3.4.4b virus virulence and transmissibility, we challenged preimmune and naive control ferrets via respiratory inhalation of aerosolized Texas/37 virus, to closely emulate natural mammalian exposure to IAV. First, we exposed a group of 3 ferrets to a low (≈3 PFU) dose of aerosolized Texas/37 virus. In agreement with prior work (*29*), we productively infected 2/3 ferrets, which led to a severe and fatal infection in serologically naive ferrets. In addition, after 48 hours of sustained contact, we detected successful transmission to cohoused naive animals in direct contact with infected animals (Table). To provide a robust challenge, both naive and preimmune ferrets received inhaled doses of 10^2.4^–10^2.5^ PFU of H5N1 virus; then, preimmune ferrets served as either donors or contacts in direct contact transmission experiments. We humanely euthanized all donor ferrets on day 3 postinoculation to assess systemic dissemination of virus.

**Figure 2:**
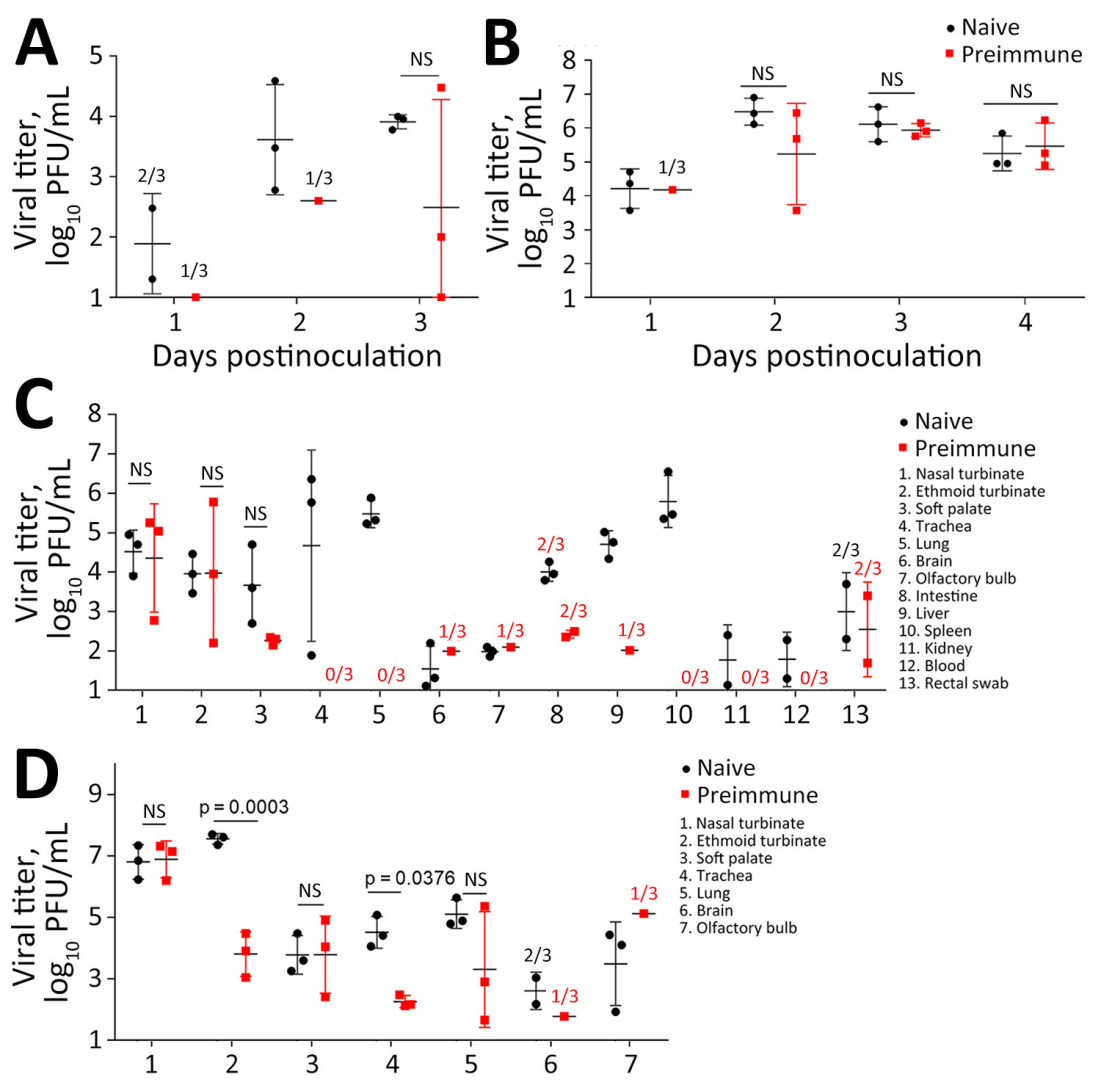
Virus shedding in study of the effect of prior influenza A(H1N1)pdm09 virus infection on pathogenesis and transmission of human influenza A(H5N1) clade 2.3.4.4b virus in ferret model. A, B) Nasal wash viral titers for influenza A(H5N1) Texas/37 virus (A) and influenza A(H7N9) Anhui/1 virus (B). C, D) Virus titers from tissues for Texas/37 H5N1 virus collected 3 days postinoculation (C) and Anhui/1 H7N9 virus collected 4 days postinoculation (D). Horizontal bars indicate median, dots indicate individual titers, whiskers indicate range of positive titers. Three naive and 3 pH1N1 preimmune ferrets were inoculated via respiratory inhalation with Texas/37 or Anhui/1 virus (Table). Nasal wash specimens (A, B) were collected daily. Virus titers were determined by standard plaque assay in MDCK cells. Tissue samples collected from nasal turbinate, ethmoid turbinate, soft palate, blood, and rectal swabs were reported in log_10_ PFU/mL. Tissues collected from lung, brain, olfactory bulb, intestines, liver, spleen, kidney were reported in log_10_ PFU/g. The limit of detection was 10 PFU per mL or g. Statistical analyses were performed using 2-way analysis of variance test when samples were positive for viral titers in all 3 inoculated animals; we considered p<0.05 statistically significant. When <3 inoculated ferrets had detectable virus, the detection frequency is indicated above the corresponding positions. Anhui/1, low pathogenicity influenza A(H7N9) A/Anhui/1/2013; NS, not statistically significant; Texas/37, highly pathogenic influenza A(H5N1) clade 2.3.4.4b A/Texas/37/2024.

**Figure 3:**
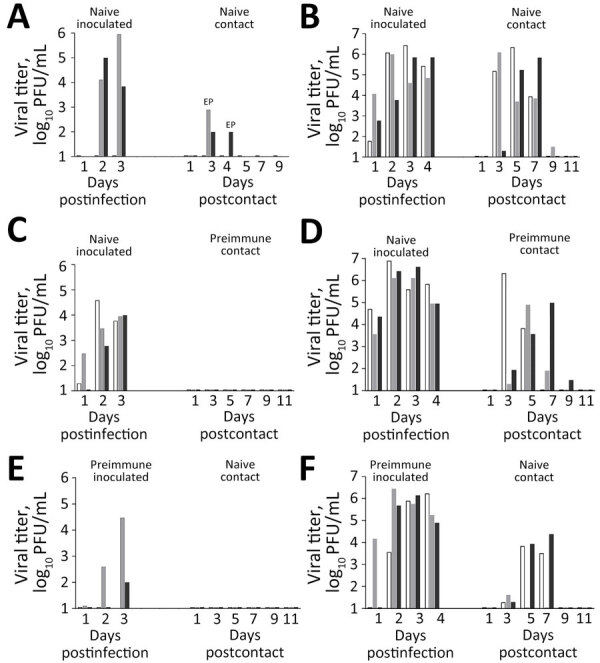
Contact transmission of Texas/37 influenza A(H5N1) virus and Anhui/1 influenza A(H7N9) virus in study of the effect of prior influenza A(H1N1)pdm09 virus infection on pathogenesis and transmission of human influenza A(H5N1) clade 2.3.4.4b virus in ferret model. A, C, E) Transmission of Texas/37 H5N1 virus among ferrets; B, D, F) transmission of Anhui/1 H7N9 virus among ferrets. Different shades indicate individual animals. We inoculated 3 naive ferrets per virus (A–D) and 3 preimmune ferrets per virus (E, F) by respiratory inhalation exposure (Table). Each inoculated ferret was pair-housed with a contact recipient, with (C, D) or without preimmunity (A, B, E, F); contact was sustained for 48 hours for Texas/37 and for 72 hours for Anhui/1 before inoculated animals were humanely euthanized. Nasal wash samples were collected daily from inoculated ferrets (days 1–4 postinfection) and on alternate days from the contact animals (days 1–11 postcontact). The limit of detection was 10 PFU/mL. Two naive contact ferrets (panel A) reached humane endpoints and were euthanized on days 3 and 4 postinfection. Anhui/1, low pathogenicity influenza A(H7N9) A/Anhui/1/2013; EP, endpoint; NS, not statistically significant; Texas/37, highly pathogenic influenza A(H5N1) clade 2.3.4.4b A/Texas/37/2024.

Serologically naive ferrets inoculated by the aerosol inhalation route with Texas/37 virus exhibited a rapid and severe infection within 3 days postinoculation (Table). As with the inoculated naive ferrets, preimmune ferrets exhibited clinical symptoms by day 3 postinoculation, but clinical signs were less severe, and lethargy, temperature rise, and weight loss were less pronounced. Virus shedding in nasal wash specimens from the preimmune ferrets was delayed (Figures 2, 3), and only 1/3 ferrets had detectable virus shedding on days 1 and 2 postinoculation, compared with 2/3 and 3/3 ferrets in the inoculated naive group at those timepoints (Figure 2, panel A). Although viral titers in day 3 postinoculation nasal wash and tissues proximal to the upper respiratory tract (i.e., nasal turbinate, ethmoid turbinate, soft palate) were comparable between the preimmune and naive ferret groups, virus was either undetectable in the lower respiratory tract (trachea, lung), extrapulmonary tissues (spleen, kidney), and blood, or detected at lower frequency in other tissues (brain, olfactory bulb, intestines, liver) in preimmune ferrets relative to naive animals (Figure 2, panel C).

Unlike the efficient transmissibility of Texas/37 virus during direct contact when both donor and contact animals were serologically naive (Table; Figure 3, panel A), Texas/37 did not transmit by that route when preimmune ferrets served as either donor or recipient animals. We did not recover infectious virus from nasal wash specimens collected from contact animals nor did those animals seroconvert to H5N1 virus (Table; Figure 3 panels C, E). Thus, we found that prior exposure to a 2019 pH1N1 virus in ferrets reduced disease severity and limited viral spread to the lower respiratory tract and extrapulmonary tissues after rechallenge with Texas/37 virus via respiratory inhalation compared with serologically naive animals. We also observed reduced transmissibility of Texas/37 virus during direct contact when either the donor or recipient animals had prior pH1N1 exposure.

### Effect of Preexisting pH1N1 Immunity on H7N9 Virus Pathogenesis and Transmission 

Because we detected antibodies against antigens other than HA and NA, such as NP, in preimmune serum samples (Figure 1), we performed a parallel experiment, to better understand the breadth and magnitude of cross-protection. For that experiment we used the group 2 low pathogenicity (H7N9 Anhui/1 virus, which has distinct HA and NA subtypes to which pH1N1 convalescent serum does not react. Anhui/1 virus has previously been reported to cause milder disease and exhibit lower replication capacity in ferrets compared with the Texas/37 virus (*11*,*22*). Donor ferrets received an aerosol inhalation dose of 10^2.6^–10^2.8^ PFU of Anhui/1 virus. Donor and contact animals cohoused for 72 hours before donor animals were humanely euthanized on day 4 postinoculation, which was a sufficient time for efficient transmission to occur in direct contact between serologically naive donor and contact animals (Table; Figure 3, panel B).

Upon Anhui/1 virus respiratory inhalation challenge, only mild clinical signs were observed in both naive and preimmune ferrets (Table). Virus shedding in nasal wash specimens and systemic tissues were generally similar across naive and preimmune donor animals, except for day 4 postinoculation ethmoid turbinate and trachea titers, which were significantly higher in naive ferrets (p<0.05) (Figure 2, panels B, D). Efficient virus transmission during direct contact was observed when preimmune ferrets served as either donor or contact animals (Figure 3, panels D, F). Overall, we found that prior exposure to pH1N1 virus had no substantial effect on H7N9 virus shedding, replication, or transmission during direct contact, in contrast to viral challenge with H5N1 virus.

## Discussion

Key elements in the risk assessment of emerging IAVs include evaluating virus transmissibility in animal models, assessing population immunity against novel IAVs, and determining disease severity and pathogenesis in humans and animals after infection (*30*). Assessing the risk for human infection and human-to-human transmission of emerging HPAI H5N1 clade 2.4.4.4b virus, which has caused sporadic human infections among poultry and dairy farm workers in the United States, is crucial for pandemic preparedness. We showed that ferrets with existing pH1N1 virus immunity had reduced disease severity and limited viral systemic spread after aerosol inhalation exposure to Texas/37 virus. Moreover, direct-contact transmission was abolished when either the donor or recipient animals had prior H1N1 immunity. However, the protective effect of pH1N1 immunity did not extend to the virus with an HA from different phylogenetic group and NA from a distinct subtype, as evidenced by the minimal effect that pH1N1 immunity had on infection and transmission after challenge with a group 2 H7N9 virus.

This study builds upon a growing body of literature on ferrets previously exposed to seasonal IAVs and subsequently challenged with either seasonal or zoonotic IAV (*19*,*21*,*31*). Use of ferrets with existing IAV immunity from prior infection with wild-type viruses can provide contextual data for risk assessment studies (*31*). Of note, we used a contemporary 2019 pH1N1 strain to establish prior immunity, which could influence the subsequent preimmune profile of animals, as supported by varying serum H1 HA and N1 NA antibody levels depending on the year of virus isolation (Figure 1). Our findings from use of 2019 pH1N1 virus underscores the importance of considering the specific pH1N1 strain used for primary infection in such studies, and conducting future work that explores the full breadth of diverse infection histories seen in humans.

Our findings extend the understanding of cross-protective effects of pH1N1 immunity against H5N1 clade 2.3.4.4b viruses by challenging animals with a recent human isolate, Texas/37. Unlike other US isolates, Texas/37 carries a PB2–627K mutation, likely contributing to enhanced virulence in ferrets (*7*). In line with a similar study using a bovine isolate (*32*), we observed reduced disease severity and systemic viral spread in pH1N1 preimmune ferrets relative to naive controls. In addition, we found that while virus shedding in nasal wash specimens was delayed in preimmune animals early (days 1–2) after infection relative to naive animals, titers from both groups reached comparable levels by day 3 postinoculation in specimens from the upper respiratory tract (nasal wash, nasal and ethmoid turbinates) and the surrounding milieu (soft palate). Of note, a previous study also reported that H1N1-preimmune ferrets exhibited substantially reduced virus shedding in nasal secretions and viral replication in both upper and lower respiratory tissues compared with naive ferrets when challenged with an H5N1 bovine isolate (*32*). The differences between that study and ours could be attributed to variation in H5N1 challenge strains, cross-protective antibody levels, or differences in inoculation doses and routes. Beyond examination of directly inoculated animals, transmission in the presence of direct contact was reduced when either donor or recipient ferrets were preimmune, likely the result of altered host susceptibility and delayed virus shedding. Although we did not assess respiratory droplet transmission in this study, we anticipate that droplet transmission would be unlikely under this more stringent transmission setting. Of note, we used inhalation of aerosolized virus to challenge the animals, which is known to delay onset of detectable virus replication and peak titers in nasal wash specimens (*24*), more closely emulating the kinetics of natural human exposure to IAVs.

In our study, using a comprehensive panel of antigens in serologic tests, we identified antibodies that reacted to H5N1 NA and the group 1 HA stalk, but not to the HA heads of H5 and H7 viruses or the group 2 HA stalk. Broadly reactive NA and HA stalk antibodies have been shown to play a key role in cross-heterosubtypic protection in animal models (*33*,*34*), which likely contributed to the cross-protection observed in our study. In addition, human epidemiologic studies have demonstrated that populations with previous exposure to pH1N1 might experience less severe outcomes from H5N1 infection (*18*). However, we only explored cross-protective immunity at approximately 3 months after seasonal pH1N1 primary infection. The duration of such immunity and correlation between the level of NA or HA stalk antibodies, specifically H5 HA stalk antibodies, and protection efficacy warrant further study, as do comprehensive evaluations of how prior vaccination may contribute to modulating disease severity. 

Our experimental evidence from the ferret model underscores the potential role of cross-reactive HA stalk and NA antibodies in reducing disease severity and transmission after H5N1 virus infection. However, future studies of larger group sizes are warranted, as are studies investigating how antibodies targeting internal influenza virus proteins, such as NP, or cellular immunity, contribute to cross-protection. In addition, a single seasonal virus infection in naive ferrets cannot fully recapitulate the complexity of human IAV immune history. Future studies involving ferrets vaccinated with different types of influenza vaccines or repeatedly exposed to seasonal viruses more closely mirroring the varied infection histories in humans will help provide deeper insights into cross-immunity elicited across different IAV subtypes. Furthermore, inclusion of multiple zoonotic strains to assess relative contributions of nonspecific effects in preimmune animals, as demonstrated here by performing tandem challenge studies and serology assessments with both H5N1 and H7N9 viruses, is a practice not typically used in the field yet shown to be a key provider of essential contextual information.

In conclusion, our results showed that prior pH1N1 virus infection more effectively reduced the replication and transmission of H5N1 virus than it did H7N9 virus in a ferret model. Those results suggest that pH1N1 virus immunity may confer some level of protection against H5N1 clade 2.3.4.4.b virus in humans.

Appendix 1 Antigens used in study of the effect of prior influenza A(H1N1)pdm09 virus infection on pathogenesis and transmission of human influenza A(H5N1) clade 2.3.4.4b virus in ferret model.

Appendix 2 Additional information on study of the effect of prior influenza A(H1N1)pdm09 virus infection on pathogenesis and transmission of human influenza A(H5N1) clade 2.3.4.4b virus in ferret model.
