# Supplementary material for: Effect of Prior Influenza A(H1N1)pdm09 Virus Infection on Pathogenesis and Transmission of Human Influenza A(H5N1) Clade 2.3.4.4b Virus in Ferret Model
Source: Emerg Infect Dis. 2025 Mar;31(3):458–66. doi: 10.3201/eid3103.241489 (PMC11878296; doi:10.3201/eid3103.241489)
Supplement: Appendix 2 — Additional information on study of the effect of prior influenza A(H1N1)pdm09 virus infection on pathogenesis and transmission of human influenza A(H5N1) clade 2.3.4.4b virus in ferret model. [file 24-1489-Techapp-s2.pdf]

Article DOI: https://doi.org/10.3201/eid3102.241498

EID cannot ensure accessibility for supplementary materials supplied by authors. Readers who have difficulty accessing supplementary content should contact the authors for assistance.

Effect of Prior Influenza A(H1N1)pdm09 Virus Infection on Pathogenesis of Human Influenza A(H5N1) Clade 2.3.4.4b Virus Isolate in Ferret Model

Appendix 2

```
Whole gene alignment NA

                  1                                                          60
Nebraska_14_2019  MNPNQKIITI GSICMTIGMA NLILQIGNII SIWVSHSIQI GNQSQIETCN KNVITYENNT
Texas_37_2024     .......T.. .....V..IV S.M....... .........T ...Y.P.P.. QSI.......
Srf res           ---------- ---------- ---------- ---------- ---------- ----------

                  61                    82                                   120
Nebraska_14_2019  WVNQTYVNIS NTNSAARQSV ASVKLAGNSS LCPVSGWAIY SKDNSVRIGS KGDVFVIREP
Texas_37_2024     ......I... ...FL.E.A. T..T...... ...I...... ....GI.... ..........
Srf res           ---------- ---------- -sss-s-sss sssss---ss s-s---s--s s-s---sss-

                  121                                                        180
Nebraska_14_2019  FISCSPLECR TFFLTQGALL NDKHSNGTIK DRSPYRTLMS CPIGEVPSPY NSRFESVAWS
Texas_37_2024     .....H.... .......... ........V. .......... ..V..A.... ..........
Srf res           ---sssssss s----s---- ssss-sssss sssss---s- -ss-s----s s-sss---s-

                  181                                                        240
Nebraska_14_2019  ASACHDGTNW LTIGISGPDS GAVAVLKYNG IITDTIKSWR NNILRTQESE CACVNGSCFT
Texas_37_2024     .......IS. .........N .......... .......... .......... ..........
Srf res           ----s-ssss -------sss --s-s--sss s-ssssssss sss-s--s-s ----ss-s--


                  241                                                        300
Nebraska_14_2019  IMTDGPSDGQ ASYKIFRIEK GKIIKSVEMK APNYHYEECS CYPDSSEITC VCRDNWHGSN
Texas_37_2024     V......N.. ......K... ..VV.....N .......... ....AGD.M. ..........
Srf res           --s--sssss s--s-ss-ss -sssssssss -sss--ss-- ---ssss-s- --s-sss---


                  301                                                        360
Nebraska_14_2019  RPWVSFNQNL EYQMGYICSG VFGDNPRPND KTGSCGPVSS NGANGVKGFS FKYGNGVWIG
Texas_37_2024     .......... ...I...... I......... G....S.MP. ...Y...... ..........
Srf res           --s-s-ssss sssss----- --s--s--ss ssssssssss s-ss-s---- -ssss-----


                  361                                                        420
Nebraska_14_2019  RTKSISSRKG FEMIWDPNGW IGTDNKFSKK QDIVGINEWS GYSGSFVQHP ELTGLDCMRP
Texas_37_2024     ....T...S. .......... TE..SS..V. ....E.TD.. .......... .....N.I..
Srf res           s-sssssss- ----s-ss-s ssssssssss s---sssss- -s------ss ssssss-s-


                  421                                               469
Nebraska_14_2019  CFWVELIRGR PEENTIWTSG SSISFCGVDS DIVGWSWPDG AELPFTIDK
Texas_37_2024     .......... .K........ ........N. .T........ .........
Srf res           ------ss-s ssssss---- ------s-ss ssssss---s sssssssss

NA ectodomain (residues 82-469) = 388
Srf res = 223


Tx37 vs Neb14 =
345/388 residues in the ectodomain were sharded (89% similarity)
NA monomer 165 buried 5A radius setting = 223 residues exposed
186/223 surface exposed residues were shared (83.4% similarity)
```

Appendix 2 Figure 1. Alignment of NA proteins of A(H1N1pdm09 Nebraska/14/2009 and A(H5N1) Texas/37/2007 viruses. The surface exposed residues (Srf res) were marked with "s" in the alignments.

```
Whole Gene alignment HA mature protein

                  1                                                              60
Texas_37_2024     DQICIGYHAN NSTEQVDTIM EKNVTVTHAQ DILEKTHNGK LCDLNGVKPL ILKDCSVAGW
Nebraska_14_2019  .TL....... ...DT...VL ........SV NL..DK.... ..K.R..A.. H.GK.NI...
Srf res           ss-s--ss-s sssss-s--s sssss-ssss s-ssssssss --s-ssssss s-ss-s---s

                  61                                                           119
Texas_37_2024     LLGNPMCDEF IRVPEWSYIV ERANPANDLC YPGSLNDYEE LKHMLSRINH FEKIQIIPK-
Nebraska_14_2019  I....E.ESL STARS..... .TS.SD.GT. ...DFIN... .REQ..SVSS ..RFE.F..T
Srf res           --s-ssssss sssss-s--- sssssssss- ssssssssss -s-s--s-ss ssssss-sss

                  120                                                           179
Texas_37_2024     SSWPNHETSL GVSAACPYQG APSFFRNVVW LIKKNDAYPT IKISYNNTNR EDLLILWGIH
Nebraska_14_2019  ......DSDK ..T....HA. .K..YK.LI. .V..GNS..K LNQT.I.DKG KEV.V.....
Srf res           ssssssssss ss-ss-ssss ssssss-s-s -sssssssss -sssss-sss ss---s----

                  180                                                           239
Texas_37_2024     HSNNAEEQTN LYKNPITYIS VGTSTLNQRL APKIATRSQV NGQRGRMDFF WTILKPDDAI
Nebraska_14_2019  .PPTIAA.ES ..Q.ADA.VF ....RYSKKF K.E....PK. RD.E...NYY ..LVE.G.K.
Srf res           s-sssss-ss s-ssssss-- ---sssssss ssss-s-sss ssss---s-s sss-sssss-

                  240                                                           299
Texas_37_2024     HFESNGNFIA PEYAYKIVKK GDSTIMKSGV EYGHCNTKCQ TPVGAINSSM PFHNIHPLTI
Nebraska_14_2019  T..AT..LVV .R..FTMERD AG.G.II.DT PVHD...T.. ..E....T.L ..Q.V..I..
Srf res           s-s-s----- -ss--sssss ss--s-s-ss ssssssss-- -ss-s-ssss ---ssssss-

                  300                                                           359
Texas_37_2024     GECPKYVKSN KLVLATGLRN SPLREKRRKR GLFGAXAGFI EGGWQGMVDG WYGYHHSNEQ
Nebraska_14_2019  .K.......T ..R....... V.SIQS---. .....I.... ....T..... ......Q...
Srf res           -sssss-sss s-s--s--ss ss-sssssss ssssss--ss ss--ss-sss ss--ssssss

                  360                                                           419
Texas_37_2024     GSGYAADKES TQKAIDGVTN KVNSIIDKMN TQFEAVGREF NNLERRIENL NKKMEDGFLD
Nebraska_14_2019  .......LK. ..N...KI.. ....V.E... ...T...K.. .H..K..... ...VD.....
Srf res           ssssssssss sss-ss-sss s-ss-sssss ssss-sssss sssssssss- ssssssssss

                  420                                                           479
Texas_37_2024     VWTYNAELLV LMENERTLDF HDSNVKNLYD KVRLQLRDNA KELGNGCFEF YHKCDNECME
Nebraska_14_2019  I......... .L.......Y .........E ...N..KN.. ..I....... ......T...
Srf res           ---ss-s-ss sss-ss--ss sssssss-ss s-sss-ss-s ssss---sss s-s-sss--s

                  480                                                           539
Texas_37_2024     SVRNGTYDYP QYSEEARLKR EEISGVKLES VGTYQILSIY STAASSLALA IMMAGLSLWM
Nebraska_14_2019  ..K....... K.....K.N. .K.D...... TRI....A.. ..V....V.V VSLGAI.F..
Srf res           s-ssssssss sssss-ssss ss-------- ---------- ---------- ----------

                  540        551
Texas_37_2024     CSNGSLQCRI CI
Nebraska_14_2019  .......... ..
Srf res           ---------- --

HA Tx37 vs Neb14 =
HA1 (1 to 329); HA2 (330 to 499)
107/238 HA1 surface residues were shared (45% similarity)
111/138 HA2 surface residues were shared (80.4% similarity)
```

Appendix 2 Figure 2. Alignment of HA proteins of A(H1N1pdm09 Nebraska/14/2009 and A(H5N1) Texas/37/2007 viruses. The surface exposed residues (Srf res) were marked with “s” in the alignments.
